# 阿帕替尼用于一线治疗进展后晚期非鳞非小细胞肺癌的疗效和生存分析

**DOI:** 10.3779/j.issn.1009-3419.2017.11.07

**Published:** 2017-11-20

**Authors:** 学敏 王, 维红 张, 伟娇 杜, 新伟 张, 秀宝 任, 水 曹

**Affiliations:** 300060 天津，天津医科大学肿瘤医院国家肿瘤临床医学研究中心，天津市“肿瘤防治”重点实验室，天津市恶性肿瘤临床研究中心，天津市肿瘤免疫与生物治疗重点实验室，生物治疗肿瘤科 National Clinical Research Center for Cancer, Tianjin Medical University Cancer Institute and Hospital; Key Laboratory of Cancer Prevention and Therapy; Tianjin Clinical Research Center for Cancer; Key Laboratory of Cancer Immunology and Biotherapy; Department of Biotherapy, Tianjin 300060, China

**Keywords:** 肺肿瘤, 阿帕替尼, 疗效, 安全性, 生存分析, Lung neoplasms, Apatinib, Efficacy, Safety, Survival analysis

## Abstract

**背景与目的:**

晚期非小细胞肺癌（non-small cell lung cancer, NSCLC）的二线、三线化疗有效率较低，靶向药物的应用为部分患者带来生存获益。阿帕替尼是一种新型小分子抗血管生成药物，在多种恶性肿瘤治疗中展现出令人满意的抗癌活性。本研究旨在评价阿帕替尼用于一线治疗进展后晚期非鳞NSCLC的安全性和疗效。

**方法:**

回顾性分析128例晚期非鳞NSCLC不同治疗组患者的疗效和生存情况，用*Kaplan-Meier*法和*Cox*模型进行分析。

**结果:**

以单纯化疗组为对照，阿帕替尼单药组、单纯化疗组和阿帕替尼联合化疗组的中位无进展生存期（progression free survival, PFS）分别为3.0个月（*P*=0.381）、3.7个月和6.0个月（*P* < 0.001），中位总生存期（overall survival, OS）分别为6.0个月（*P*=0.494）、6.5个月和9.0个月（*P*=0.001）。3级-4级不良反应发生率分别为18.5%、15.8%和16.0%（*P*=0.947）。治疗方案（*P*=0.018）及体能状态（performance status, PS）（*P* < 0.001）是PFS的独立影响因素，吸烟史（*P*=0.014）、治疗方案（*P*=0.002）和PS（*P* < 0.001）是OS的独立影响因素。

**结论:**

阿帕替尼安全性高，肺癌一线治疗失败后，二线或三线化疗联合阿帕替尼，与单纯化疗相比，患者有PFS和OS获益，阿帕替尼单药与单纯化疗组间PFS和OS无明显差异；无吸烟史、PS 0分-1分和联合治疗的患者预后更好。

## Abstract (en)

**Background and objective:**

The efficacy of second or third-line chemotherapy in advanced non-small cell lung cancer (NSCLC) patients is low. The use of targeted drugs brings survival benefit for some patients. Apatinib, as a novel small molecule antiangiogenic drug, has demonstrated satisfactory anticancer activity across a broad range of malignancies. The aim of this study is to evaluate the efficacy and safety of apatinib in patients with advanced NSCLC after first-line treatment failure.

**Methods:**

A retrospective study of 128 patients was conducted to evaluate the safety, short-term efficacy and survival status with different regimens. *Kaplan-Meier* method and *Cox* regression model were used for analysis.

**Results:**

Compared with chemotherapy alone, the median progression free survival (PFS) in apatinib monotherapy, chemotherapy alone and apatinib combined with chemotherapy were 3.0 (*P*=0.381), 3.7 and 6.0 months (*P* < 0.001), respectively. The median overall survival (OS) were 6.0 (*P*=0.494), 6.5 and 9.0 months (*P*=0.001), respectively. The incidence of adverse events in grades 3-4 were 18.5%, 15.8% and 16.0%, respectively (*P*=0.947). Different treatment regimens (*P*=0.018) and performance status (PS)(*P* < 0.001) were the independent factors of PFS. The smoking history (*P*=0.014), treatment regimens (*P*=0.002) and PS (*P* < 0.001) were independent influencing factors of OS.

**Conclusion:**

Apatinib has a good security. After first-line treatment failure of lung cancer, chemotherapy combined with apatinib in second or third-line is beneficial in PFS and OS when compared with chemotherapy alone. But when making comparison between apatinib monotherapy and chemotherapy alone, there is no significant difference in PFS and OS. Patients who never smoke or has a better PS or use combination therapy have longer survival time.

肺癌是全球癌症相关死亡的首要原因，5年生存率仅约20%^[1]^，其中非小细胞肺癌（non-small cell lung cancer, NSCLC）占85%，且56%患者确诊时已发生不同程度的转移^[2]^。肺癌高发病率、高死亡率等特点使其成为全球关注的重要公共健康问题。目前一线标准化疗是含铂类两药联合化疗，但很多患者因耐药更换方案，但二线、三线化疗有效率低，失败后无确切方案^[3]^。因此，急需探索出更有效的治疗为患者带来生存获益。

靶向药物旨在作用于肿瘤形成过程的相关分子，与传统化疗药相比因靶点明确、不良反应少而成为近年研究热点^[4]^。对于有基因突变患者，可明显延长生存时间和改善生活质量^[5]^。抗血管生成药是靶向药物重要组成部分。肿瘤血管生成为增殖细胞提供氧气和营养物质，引起肿瘤进展和转移^[6]^。基于这一理论，抗血管生成可达到抗肿瘤目的。

阿帕替尼（艾坦，YN968D1）是一种新型口服小分子抗血管生成药^[7]^，在多种实体瘤治疗中效果明显^[8]^。李旭等^[9]^研究发现：与紫杉醇对比，阿帕替尼可显著降低肺癌患者的血清癌胚抗原（carcino-embryonic antigen, CEA）和血管内皮生长因子（vascular endothelial growth factor, VEGF）水平（*P* < 0.05）。有研究^[10]^得出：阿帕替尼与化疗联合应用抑瘤效应最强。目前关于阿帕替尼治疗晚期肺癌的临床研究极少，故进行本回顾性研究，以评价阿帕替尼治疗晚期非鳞NSCLC的安全性和疗效。

## 1 资料与方法

### 1.1 临床资料

收集2015年6月-2017年2月在天津医科大学肿瘤医院就诊的NSCLC患者，纳入标准：①年龄≥18岁；②经病理学确诊为非鳞NSCLC；③临床分期Ⅲb期或Ⅳ期且一线治疗后进展；④后续治疗与之前方案间隔至少4周；⑤体能状态（performance status, PS）0分-2分；⑥未接受其他抗血管生成药且一线治疗失败后未应用靶向药；⑦至少1个可测量靶病灶且靶病灶未经过放疗；⑧阿帕替尼服用≥2个月，起始剂量为500 mg/d；化疗药应用≥2个周期，治疗方案应用直至疾病进展或出现不可耐受的不良反应。经筛选共128例患者纳入本研究，依治疗方案分为3组：阿帕替尼单药组27例（21.1%）、单纯化疗组76例（59.4%）和阿帕替尼联合化疗组25例（19.5%）。共57例患者治疗前有明确的基因检测，基因突变阴性者34例（59.6%），阳性者23例（40.4%），包括*EGFR*突变（19例）、*c-Met*扩增（1例）和*K-ras*突变（3例）。一线治疗方案包括靶向药物表皮生长因子受体酪氨酸激酶抑制剂（epithelial growth factor receptor tyrosine kinase inhibitors, EGFR-TKIs）和含铂类两药联合化疗。3组共18例患者一线应用EGFR-TKIs，包括厄洛替尼（2例）、吉非替尼（4例）和埃克替尼（12例）；化疗药物包括：培美曲塞、紫杉醇脂质体、多西他赛、吉西他滨和长春瑞滨。二线化疗药物主要为多西他赛和培美曲塞。三组临床特征均无统计学差异（表 1）。

**1:** 三组不同治疗方案组患者临床特征[*n* (%）] The clinical characteristics of patients among three different treatment regimens [*n* (%)]

Variable	Total	Apatinib	Chemotherapy	Apatinib and chemotherapy	*P*
Gender					0.097
Male	87 (68.0)	23 (85.2)	48 (63.2)	16 (64.0)	
Female	41 (32.0)	4 (14.8)	28 (36.8)	9 (36.0)	
Performance status					0.089
0-1	60 (46.8)	8 (29.6)	41 (53.9)	11 (44.0)	
2	68 (53.2)	19 (70.4)	35 (46.1)	14 (56.0)	
Age (yr)					0.115
< 60	55 (43.0)	7 (25.9)	35 (46.1)	13(52.0)	
> 60	73 ((57.0)	20 (74.1)	41 (53.9)	12(48.0)	
Smoking history					0.105
Yes	82 (64.1)	22 (81.5)	45 (59.2)	15(60.0)	
No	46 (35.9)	5 (18.5)	31 (40.8)	10(40.0)	
Histology					0.317
Adenocarcinoma	124 (96.9)	25(92.6)	75 (98.7)	24 (96.0)	
Large cell lung cancer	4 (3.1)	2 (7.4)	1 (1.3)	(4.0)	
Staging					0.279
Ⅲb	16(12.5)	4 (14.8)	11 (14.5)	(4.0)	
Ⅳ	112 (87.5)	23 (85.2)	65 (85.5)	24 (96.0)	
Surgical history					0.098
Yes	26 (20.3)	9 (33.3)	11 (14.5)	6(24.0)	
No	102 (79.7)	18 (66.7)	65 (85.5)	19(76.0)	
Gene mutation					0.304
Positive	23 (18.0)	7 (25.9)	14(18.4)	2(8.0)	
Negative	34 (26.6)	9 (33.3)	18 (23.7)	7(28.0)	
Unknown	71 (55.4)	11 (40.8)	44 (57.9)	16(64.0)	
Treatment stage					0.434
Second-line	57 (44.5)	11 (40.7)	32 (42.1)	14(56.0)	
Third-line	71 (55.5)	16 (59.3)	44 (57.9)	11(44.0)	

### 1.2 近期疗效评价

应用阿帕替尼1个月或化疗2周期后评价，根据实体瘤疗效评价标准（Response Evaluation Criteria in Solid Tumors, RECIST）分为完全缓解（complete response, CR）、部分缓解（partial response, PR）、疾病稳定（stable disease, SD）和疾病进展（progressive disease, PD）。客观反应率（objective response rate, ORR）=（CR+PR）/（CR+PR+SD+PD）×100%；疾病控制率（disease control rate, DCR）=（CR+PR+SD）/（CR+PR+SD+PD）×100%。

### 1.3 随访和生存分析

采用电话随访，末次随访时间为2017年4月10日。无进展生存期（progression-free survival, PFS）定义为患者自治疗开始至明确为PD的时间；总生存期（overall survival, OS）为自患者治疗开始至死亡或末次随访时间。

### 1.4 不良反应评价

根据不良事件常用术语标准（Common Terminology Criteria for Adverse Events, CTC AE 4.0）对不良反应进行统计（1级-4级）。

### 1.5 统计学分析

采用SPSS 22.0软件进行统计学分析。用卡方检验对临床特征、近期疗效进行比较；用非参数检验对不良反应进行比较；用*Kaplan-Meier*法和*Cox*回归模型对PFS和OS进行分析。*P* < 0.05为差异有统计学意义。

## 2 结果

### 2.1 疗效评价

128例患者疗效均可评价。阿帕替尼单药组、单纯化疗组和阿帕替尼联合化疗组均无CR患者，达PR者分别为1例、2例和2例（图 1），达SD分别为10例、32例和15例，三组间ORR（*χ*^2^=1.238, *P*=0.538）和DCR（*χ*^2^=4.888, *P*=0.087）差异均无统计学意义（表 2）。化疗联合阿帕替尼组与单纯化疗相比，中位PFS（总体：6.0个月和3.7个月，*P* < 0.001；二线：8.0个月和4.0个月，*P*=0.027；三线：5.0个月和3.6个月，*P*=0.004）和OS（总体：9.0个月和6.5个月，*P*=0.001；二线：11.0个月和8.3个月，*P*=0.017；三线：9.0个月和6.0个月，*P*=0.033）差异均有统计学意义。阿帕替尼单药与单纯化疗组比较，中位PFS（总体：3.0个月和3.7个月，*P*=0.381；二线：4.6个月和4.0个月，*P*=0.221；三线：3.0个月和3.6个月，*P*=0.803）和OS（总体：6.0个月和6.5个月，*P*=0.494；二线：7.0个月和8.3个月，*P*=0.159；三线：4.5个月和6.0个月，*P*=0.575）差异均无统计学意义（图 2）。

**1:**
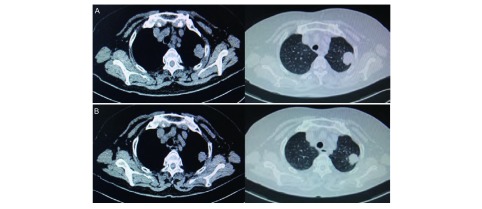
肺腺癌患者安某胸CT。A：服用阿帕替尼前；B：服用阿帕替尼3个月后。 The thoracic spiral CT of lung adenocarcinoma patient. A: Before using apatinib; B: Using apatinib three months later. CT: computed tomography.

**2:** 三组不同治疗方案组近期疗效 The short-term efficacy comparison among three different treatment regimens

Group	*n*	CR	PR	SD	PD	ORR	DCR
Apatinib	27	0	1	10	16	3.7%	40.7%
chemotherapy	76	0	2	32	42	2.6%	44.7%
Apatinib and chemotherapy	25	0	2	15	8	8.0%	68.0%
CR: response rate; PR: partial response; SD: stable disease; PD: progressive disease; ORR: objective response rate; DCR: disease control rate.							

**2:**
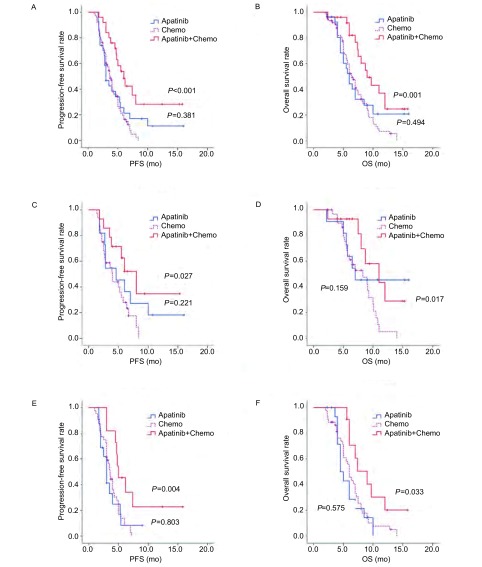
阿帕替尼联合化疗*vs*单纯化疗、阿帕替尼单药*vs*单纯化疗的PFS/OS曲线。A：总体PFS曲线；B：总体OS曲线；C：二线治疗PFS曲线；D：二线治疗OS曲线；E：三线治疗PFS曲线；F：三线治疗OS曲线。 The PFS/OS curves between apatinib combined with chemotherapy and chemotherapy alone, between apatinib and chemotherapy alone. A: overall PFS curves; B: overall OS curves; C: PFS curves of second-line treatment; D: OS curves of second-line treatment; E: PFS curves of third-line treatment; F: OS curves of third-line treatment.

### 2.2 不良反应

128例患者均可评估毒副反应，不良反应发生后予对症处理。5例患者调整阿帕替尼剂量为250 mg/d：阿帕替尼单药组和联合化疗组分别为11.1%（3/27）和8.0%（2/25）。3组均未出现因不良反应死亡事件，3级-4级不良反应发生率分别为18.5%（5/27）、15.8%（12/76）和16.0%（4/25），差异无统计学意义（*P*=0.947）（表 3）。

**3:** 三组不同治疗方案组不良反应比较 The toxicities comparison among three different treatment regimens

Toxicity	Apatinib (*n*=27)			Chemotherapy (*n*=76)			Apatinib and chemotherapy (*n*=25)			*P*
	1-2	3-4		1-2	3-4		1-2	3-4		
Hypertension	13	3		5	0		14	2		0.566
Hand-foot syndrome	12	1		3	0		11	2		0.679
Proteinuria	5	0		7	1		3	0		0.607
Bone marrow suppression	2	0		32	10		4	0		0.413
Vomiting/Diarrhea	10	1		18	0		8	0		0.307
Liver damage	2	0		4	1		1	0		0.741

### 2.3 预后因素分析

治疗方案（HR=0.687, *P*=0.018）及PS（HR=3.220, *P* < 0.001）是PFS的独立影响因素；吸烟史（HR=0.410, *P*=0.014）、治疗方案（HR=0.589, *P*=0.002）及PS（HR=2.241, *P* < 0.001）是OS的独立影响因素（表 4）。

**4:** *Cox*多因素分析 *Cox* multivariate analysis

Variable	PFS			OS	
	HR（95%CI）	*P*		HR（95%CI）	*P*
Gender	0.849（0.411-1.753）	0.658		0.657（0.319-1.352）	0.254
Performance status	3.220（2.074-4.998）	< 0.001		2.241（1.454-3.453）	< 0.001
Age	0.996（0.976-1.016）	0.661		0.999（0.979-1.020）	0.944
Smoking history	0.610（0.300-1.243）	0.173		0.410（0.201-0.837）	0.014
Histology	1.511（0.895-2.553）	0.123		1.573（0.932-2.654）	0.090
Staging	1.501（0.750-3.004）	0.251		1.832（0.876-3.831）	0.108
Surgical history	0.611（0.351-1.066）	0.185		0.738（0.424-1.287）	0.285
Treatment regimens	0.687（0.504-0.938）	0.018		0.589（0.425-0.817）	0.002

## 3 讨论

VEGF及其受体（VEGFRs）在多种肿瘤血管内皮及淋巴管高度表达^[11]^，为肿瘤治疗提供了新靶点。VEGFR2是VEGF介导的血管生成通路的主要介质^[12]^，多种因素引起VEGF产生增多后刺激VEGFR-2发生自体磷酸化，引发肿瘤的血管生成、增殖和转移^[7]^。

目前肺癌治疗应用的抗血管生成药主要有针对VEGF的单克隆抗体——贝伐珠单抗（Bevacizumab），ECOG 4599研究^[13]^中：非鳞NSCLC患者一线接受紫杉醇-卡铂联合贝伐珠单抗，与单纯化疗相比，有明显的PFS（6.2 mo *vs* 4.5 mo, *P* < 0.001）和OS（12.3 mo *vs* 10.3 mo, *P*=0.003）获益，但治疗相关死亡风险增加，且年老者（≥70岁）不能从中获益。另一获批准用于NSCLC的药物为重组人血管内皮抑素（恩度），Ⅲ期临床研究^[14]^表明：长春瑞滨-铂类联合重组人血管内皮抑素与对照组相比可显著延长患者中位肿瘤进展时间（6.3 mo *vs* 3.6 mo, *P* < 0.001）。

阿帕替尼作用靶点主要为VEGFR-2，抑制VEGF与之结合及自体磷酸化，从而抑制血管形成，降低肿瘤微血管密度^[7]^。此外，阿帕替尼还能减少肿瘤细胞对化疗药的耐药^[15]^。晚期胃癌的Ⅲ期临床研究^[16]^表明：与对照组相比，阿帕替尼可延长患者中位OS（195 d *vs* 140 d, *P*=0.015, 6），2014年我国批准其用于晚期胃癌治疗。张力等^[17]^进行的肺癌Ⅱ期临床研究表明：阿帕替尼与对照组相比可显著延长晚期非鳞NSCLC患者中位PFS（4.7 mo *vs* 1.9 mo, *P* < 0.000, 1）。

目前临床上阿帕替尼治疗肺癌的方案包括单用或与化疗联用，本研究中27例患者单用该药，因该组患者年龄偏大（≥60岁占74.1%），体能状态相对较差（PS=2占70.4%），阿帕替尼作为三线治疗比例大（占59.3%），故虽然阿帕替尼单药并非为可耐受化疗患者的常规方案，结合上述情况考虑纳入该部分患者作为研究对象以评价单药疗效。

肺癌二线化疗药物多西他赛/培美曲塞单药的ORR约为10%^[18]^。本研究近期疗效分析得出阿帕替尼单药组、单纯化疗组和阿帕替尼联合化疗组的ORR分别为3.7%、2.6%和8.0%，低于常规化疗，考虑与本研究纳入大部分三线治疗患者（55.5%）、样本数量小、PS评分偏高（PS=2占53.2%）及大数据临床研究可能存在样本选择偏倚有关。

本研究首次将阿帕替尼的不同应用方案与常规化疗进行比较，结果表明：一线治疗失败后，以单纯化疗组为对照，化疗联合阿帕替尼的患者有PFS（总体：6.0个月和3.7个月，*P* < 0.001；二线：8.0个月和4.0个月，*P*=0.027；三线：5.0个月和3.6个月，*P*=0.004）和OS（总体：9.0个月和6.5个月，*P*=0.001；二线：11.0个月和8.3个月，*P*=0.017；三线：9.0个月和6.0个月，*P*=0.033）获益，与王永莎等^[10]^的实验结论一致。并且无论二线或三线治疗时在化疗基础上联合应用阿帕替尼，患者均有生存期的延长。联合应用时临床疗效更佳，除了抗肿瘤效应叠加外，还可能与阿帕替尼增强机体对化疗药敏感性，逆转化疗耐药性而提高疗效相关^[15]^。但因样本量小，尚无法分析阿帕替尼联合何种化疗药可达到最佳抗瘤效应。而阿帕替尼单药组与单纯化疗组比较，患者PFS（总体：3.0个月和3.7个月，*P*=0.381；二线：4.6个月和4.0个月，*P*=0.221；三线：3.0个月和3.6个月，*P*=0.803）和OS（总体：6.0个月和6.5个月，*P*=0.494；二线：7.0个月和8.3个月，*P*=0.159；三线：4.5个月和6.0个月，*P*=0.575）差异均无统计学意义，提示单独服用阿帕替尼与同期单纯化疗的效果无明显差异，该结果与张力等^[17]^研究结论不符，考虑与本研究为回顾性研究且两项研究患者入选标准、阿帕替尼应用剂量、应用时期和对照组化疗药种类不同有关。

多因素分析表明化疗联合阿帕替尼可延长患者PFS（*P*=0.018）和OS（*P*=0.002），与单因素分析结果基本一致；PS 0分-1分患者有PFS（*P* < 0.001）和OS（*P* < 0.001）获益，与Girard等^[19]^的研究结论相符，提示PS好的患者应积极进行后续治疗。

在3级-4级不良反应方面，三组无明显差异。阿帕替尼的毒性反应主要包括高血压、蛋白尿、手足综合征和消化道反应，但多为轻中度，可耐受，不能耐受者通过减量或停药可控制^[17]^。表明该药具有良好的安全性。

确切的分子标志物有利于肿瘤精准化治疗的进行。阿帕替尼治疗乳腺癌的生物标记物研究表明：VEGFR-2过度表达和高血压是提高乳腺癌临床有效率和延长PFS的独立影响因素^[20]^。王博等^[21]^研究提示：服用阿帕替尼的胃癌患者中，甲胎蛋白（*α*-fetoprotein, AFP）阳性者疾病控制及生存获益优于AFP阴性者。但目前临床上尚无恰当的分子标志物可对肺癌患者服用阿帕替尼的最佳受益人群进行筛选。

本研究得出了关于阿帕替尼治疗晚期非鳞NSCLC较为满意的结论，但存在局限性，比如是回顾性研究且样本量较小，故结论需前瞻性研究和大样本资料进一步验证，此外以下问题尚未解决：①阿帕替尼联合何种化疗药效果最佳；②分子标志物的筛选。目前正在开展阿帕替尼治疗肺癌、乳腺癌和肝癌的Ⅲ期临床研究。期待这些研究能让我们对阿帕替尼有更深刻的认识。
